# Crystal Structures of the Putative Isocitrate Dehydrogenase from *Sulfolobus tokodaii* Strain 7 in the Apo and NADP^+^-Bound Forms

**DOI:** 10.1155/2018/7571984

**Published:** 2018-12-19

**Authors:** Hisanori Kondo, Midori Murakami

**Affiliations:** Department of Physics, Graduate School of Science, Nagoya University, 464-8602 Nagoya, Japan

## Abstract

Isocitrate dehydrogenase is a catabolic enzyme that acts during the third step of the tricarboxylic acid cycle. The hypothetical protein ST2166 from the archaeon *Sulfolobus tokodaii* was isolated and crystallized. It shares high primary structure homology with prokaryotic NADP^+^-dependent IDHs, suggesting that these enzymes share a common enzymatic mechanism. The crystal structure of ST2166 was determined at 2.0 Å resolution in the apo form, and then the structure of the crystal soaked with NADP^+^ was also determined at 2.4 Å resolution, which contained NADP^+^ bound at the putative active site. Comparisons between the structures of apo and NADP^+^-bound forms and NADP-IDHs from other prokaryotes suggest that prokaryotic NADP-IDHs recognize their cofactors using conserved Lys335, Tyr336, and Arg386 in ST2166 at the opening cleft before the domain closure.

## 1. Introduction

Isocitrate dehydrogenase (IDH) catalyzes the oxidative decarboxylation of isocitrate to produce *α*-ketoglutarate and CO_2_ while reducing the cofactor nicotinamide adenine dinucleotide (phosphate) (NAD(P)^+^) to NAD(P)H using divalent metal cation (Mg^2+^ or Mn^2+^) in the tricarboxylic acid cycle. According to the cofactor specificity, two isozymes with IDH activity are known, NAD^+^-dependent IDH (EC 1.1.1.41) and NADP^+^-dependent IDH (NADP-IDH; EC 1.1.1.42) [1–3]. NADP-IDHs can be mainly divided into two subfamilies based on the sequence similarity; subfamily I contains prokaryotic IDHs including archaeal and most bacterial IDHs, while subfamily II includes eukaryotic and some bacterial IDHs [4]. Although the amino acid sequence identity between subfamilies I and II is low (<20%), both IDHs function as homodimers; each monomer is composed of three domains: large, small, and clasp domains, and the active site is situated between the large and small domains.

IDH from *Escherichia coli* (EcIDH) belonging to subfamily I has been studied most extensively. The crystal structures of the enzyme have been solved in different enzymatic states with the substrate/product. The apo EcIDH can adopt both open and closed conformations, [2, 5]. Upon binding of the substrate/product to the active site, large (~20°) rotation of the large domain is induced with respect to the small and clasp domains [6–9]. The catalytic activity of EcIDH is regulated by bifunctional enzyme IDH kinase/phosphatase (AceK) by phosphorylation/dephosphorylation of Ser113 in the loop (called “phosphorylation loop”) near the active site [2, 10]. Recent studies show that the enzymatic activity of EcIDH is also controlled by reversible acetylation of lysine residues near the isocitrate binding site and at the molecular surface [11, 12].

All complex structures of EcIDH containing NADP^+^ adopt closed conformations. The structures of other prokaryotic NADP-IDHs have been reported, in which IDHs bind to NADP^+^ in a closed conformation similar to that of EcIDH with NADP^+^, whereas IDH from the hyperthermophile *Aeropyrum pernix* (ApIDH) could bind to NADP^+^ and isocitrate even though their active sites are open [13, 14]. These structures exhibit separate binding sites for isocitrate and NADP^+^, suggesting that prokaryotic NADP-IDHs use a random-ordered binding scheme to bind the substrate and cofactor [15–17]. However, among these structures, their putative residues for binding with cofactors, especially with 2′-phosphate, are not completely identical.

The nicotinamide ring of NADP^+^ is not visible in almost conformations of prokaryotic NADP-IDHs complexed with NADP^+^, suggesting that the cofactor is hydrolyzed in the presence of isocitrate during the crystallization process. Because no crystal structures of EcIDH complexed with only NADP^+^ in the open form have been available [18], further structural information and insights are still needed to elucidate how and when prokaryotic NADP-IDHs recognize their cofactor accurately.

The thermoacidophilic archaeon *Sulfolobus tokodaii* strain 7, which grows optimally at 75°C and pH 3, has an open reading frame ST2166 in the genome encoding hypothetical IDH [19]. The monomer contains 409 amino acid residues with a molecular weight of 46,492 with the consensus sequence of IDH, and the amino acid sequence shares 50.2% and 48.9% identities with ApIDH and EcIDH, respectively. In this study, we expressed, purified, and determined the crystal structures of ST2166 in the apo and NADP^+^-bound forms at 2.0 Å and 2.4 Å resolutions, respectively, and discussed the mechanisms for cofactor recognition of prokaryotic NADP-IDHs.

## 2. Materials and Methods

### 2.1. Purification of the Recombinant Proteins

ST2166 was produced and purified according to the routine protocol [20]. *E. coli* (Rosetta-gami DE3) transformed with the plasmid pST2166, which was the gift from Professor S. Kuramitsu (Osaka University), was grown at 37°C in the 2X YT medium containing 16 g/L Bacto tryptone, 10 g/L Bacto yeast extract, and 5 g/L NaCl. The cells were harvested by centrifugation at 8000 ×g for 5 min, washed with a Tris buffer (20 mM Tris-HCl (pH 8.5), 1 mM EDTA, 1 mM 2-mercaptoethanol, and 1 mM PMSF). The harvested cells were disrupted by sonication using an ultrasonic homogenizer (150 W). Cell debris and large particles were removed by centrifugation at 40,000 ×g for 30 min. The supernatant fraction was incubated at 90°C for 10 min for the denaturation of intrinsic proteins of *E. coli*, and their denatured proteins were removed by centrifugation at 40,000 ×g for 30 min at 4°C. Residual soluble proteins from *E. coli* in the supernatant were precipitated in 60% (*w*/*v*) of ammonium sulfate in Tris buffer with stirring for 3 h at 4°C and then were removed by centrifugation at 40,000 ×g for 30 min. The supernatant was applied to a 25 mL affinity column (Red-TOYOPEARL) equilibrated with the Tris buffer. Proteins were eluted with a linear gradient of 0.1–0.8 M NaCl in the Tris buffer. Fractions containing ST2166 were pooled, dialyzed against the Tris buffer, and then subjected to an anion exchange chromatography using a tandem of three HiTrap Q HP columns equilibrated with the Tris buffer. Proteins were eluted with a linear gradient of 0–1.0 M NaCl in the Tris buffer. The pooled fractions containing ST2166 were dialyzed against 20 mM Tris-HCl (pH 7.5) containing 1 mM 2-mercaptoethanol and concentrated with Centriprep-30 (Amicon).

### 2.2. Crystallization

For the crystallization of ST2166 in the apo form, the recombinant protein solution (8 mg/mL) was used. Crystals were obtained by the hanging-drop vapor diffusion method against the crystallization solution containing 13% PEG 10,000 and 100 mM HEPES (pH 7.5) at 10°C. The final conditions employed a 3 *μ*L drop containing 2 *μ*L of protein solution and 1 *μ*L of crystallization solution. Pilar-shaped crystals grew in dimensions of 1 × 0.1 × 0.01 mm in few days.

For cocrystallization with NADP^+^, the apo crystals were soaked with 25 mM NADP^+^ in the crystallization solution (soaking solution) at 10°C for 24 h.

### 2.3. Diffraction Data Collection

Crystals were soaked for 10 min in the crystallization or soaking solution containing 30% (*v*/*v*) sucrose as a cryoprotectant. Subsequently, they were flash-cooled with liquid nitrogen. During the data collection, the crystal was kept at 100 K under a gas flow of cold nitrogen from a cryostream. Diffraction images were collected using a CCD detector (ADSC Quantum 4) at the beamline BL38B1 of SPring-8 (Harima, Japan). Diffraction data were processed with Mosflm [21], Scala [22], and Truncate [23] incorporated in the CCP4 program suite [24].

### 2.4. Structural Analysis

The crystal structure of ST2166 was solved by the molecular replacement method using the open form of ApIDH (PDB ID: 1V94) as an initial model; then, crystallographic refinements were executed using CNS1.0 [25]. Manual model building was performed with XtalView [26]. Subsequently, the resulting structure of apo ST2166 was used as an initial model for the complex structure with NADP^+^. The qualities of the final models were assessed by using the program PROCHECK [27]. The statistics for data collection and refinements were summarized in Table 1.

Coordinates and structural parameters had been published with Protein Data Bank under accession code 2E0C for the apo form and 2E5M for the NADP^+^-bound form.

Figures were generated with Clustal W [28], ESPript [29], and PyMOL [30].

## 3. Results and Discussion

### 3.1. Overall Structure of the Apo Form of ST2166

ST2166 crystallized in a monoclinic crystal belonging to P2_1_ that diffracted X-rays up to 2.0 Å resolution. In this crystal, the asymmetric unit contains two monomers having nearly identical overall structures with root-mean-square deviation (RMSD) for least-squares fitting of C*α* atoms of 0.18 Å. The final model comprises 401 amino acid residues per subunit and 463 water molecules. Residues 101–108, part of the Cd loop and d helix (residues 97–118) equivalent to the phosphorylation loop in EcIDH, are disordered.

**Figure 1:**
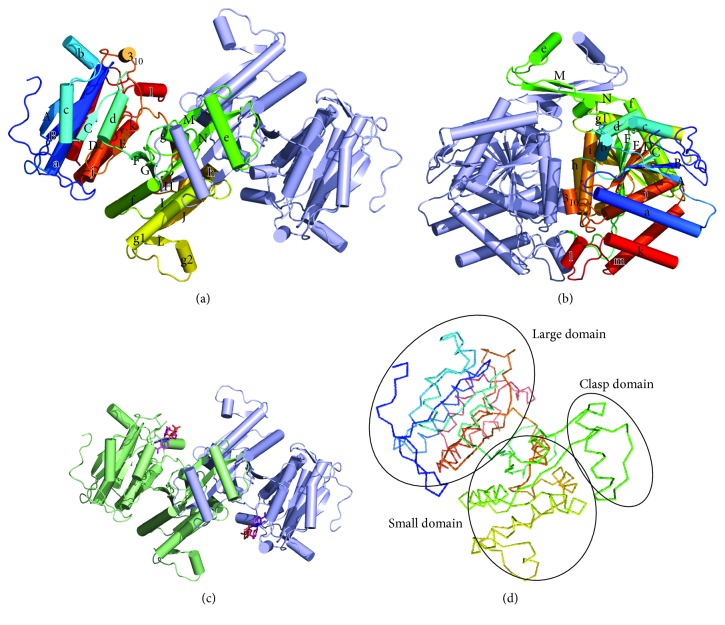
Overview of structures of homodimer of ST2166. (a) Structure of the apo form. The view is along a noncrystallographic 2-fold axis, which relates the two subunits of the dimer. Polypeptide chains are shown with a cylinder model and colored rainbow from blue (N-terminus) to red (C-terminus) in A chain and light blue in B chain, respectively. (b) View of the monomer of the apo form. Polypeptide chain is shown with a ribbon model. (c) Structure of the NADP^+^-bound form. NADP^+^ molecule is drawn as a line model. Oxygen, nitrogen, and sulfur atoms are shown in purple, red, blue, and orange, respectively. (d) View of the monomer of the apo form drawn in a ribbon model.

Figures 1(a) and 1(b) show the dimer structure of ST2166 of the apo form. Each subunit containing 17 *α*-helices and 15 *β*-strands is subdivided into the large domain (residues 1–120 and 302–409), the small domain (residues 121–153 and 196–301), and the clasp domain (residues 154–195) (Figure 1(d)).

**Figure 2:**
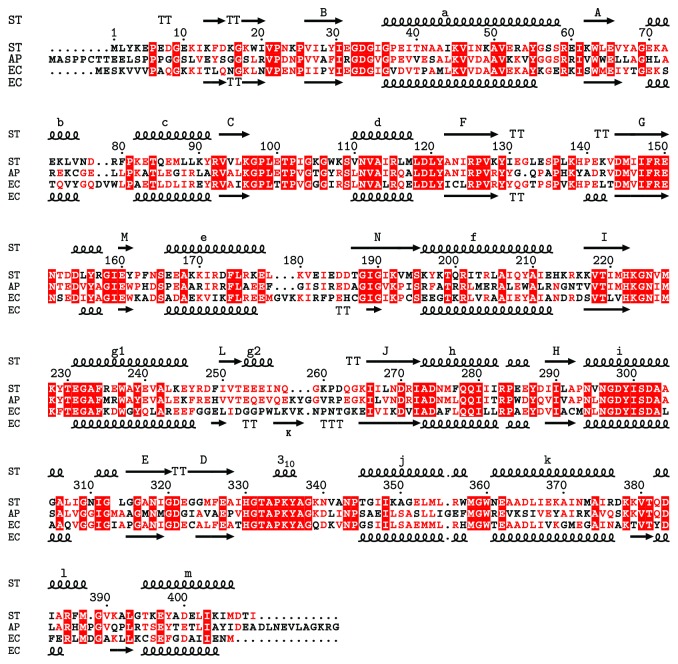
Multiple alignment of sequences for ST2166, ApIDH, and EcIDH. The secondary structure elements were indicated and labeled as helical lines and A to K for *α*-helices and as solid arrows and a to m for *β*-strands, whose nomenclature was proposed by Hurley et al. [2]. Identical residues were shaded in red; similar residues were colored in red.

Figure 2 shows the sequence alignment of ST2166, ApIDH, and EcIDH. Their secondary structures are well conserved with RMSD of 1.4 Å and 2.7 Å for 400 and 396 C*α* atoms between ST2166 and the open forms of ApIDH and between ST2166 and EcIDH, respectively. Noticeable differences are seen preferentially in the small domain. The g2 helix in the small domain of ST2166 is shared with ApIDH, but not EcIDH with the K strand forming there. Although the L strand is conserved among them, it goes in the opposite direction in EcIDH to that in ST2166 and ApIDH.

### 3.2. Active Site

**Figure 3:**
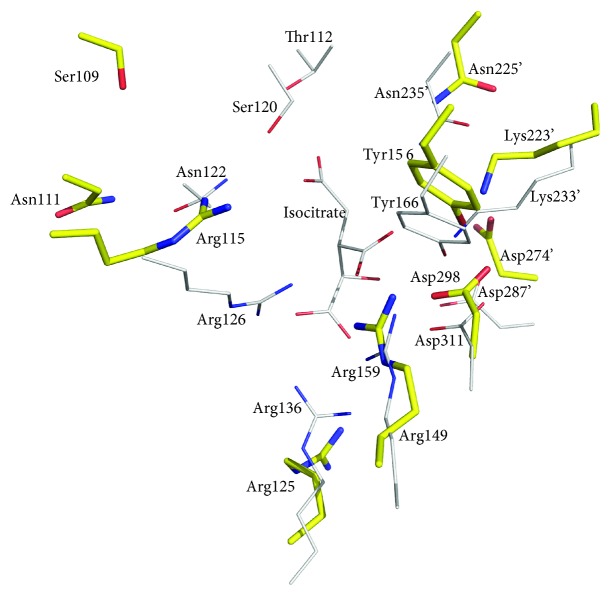
Superimposition of the putative isocitrate binding site of ST2166 (yellow stick) and the closed form of ApIDH (white line). Oxygen and nitrogen atoms are shown in red and blue, respectively.

In the structure of the apo form, putative isocitrate-binding residues (Ser109, Asn111, Arg115, Arg125, Arg149, Asp298, Tyr156, Lys223′, Asn225′, and Arg274′, the prime designates a residue from the adjacent subunit) involved in the binding of isocitrate in prokaryotic NADP-IDHs are exposed to the inner surface in the deep cleft of the active site (Figure 3). Therefore, it is probable that ST2166 has the same catalytic mechanism as proposed for other prokaryotic NADP-IDHs.

### 3.3. Overall Structure of the NADP^+^-Bound Form

When the apo crystals were soaked with NADP^+^, ST2166 could bind NADP^+^ within the active site without conformational changes with RMSD of 0.19 Å for 401 C*α* atoms between those in the apo and NADP^+^-bound (holo) forms (Figures 1(a) and 1(c)). The crystal structure in complex with NADP^+^ contains 403 amino acid residues (residues 103–108 are disordered) per subunit, NADP^+^, and 405 water molecules. The secondary structures are also completely conserved as in the apo form.

### 3.4. Active Site

**Figure 4:**
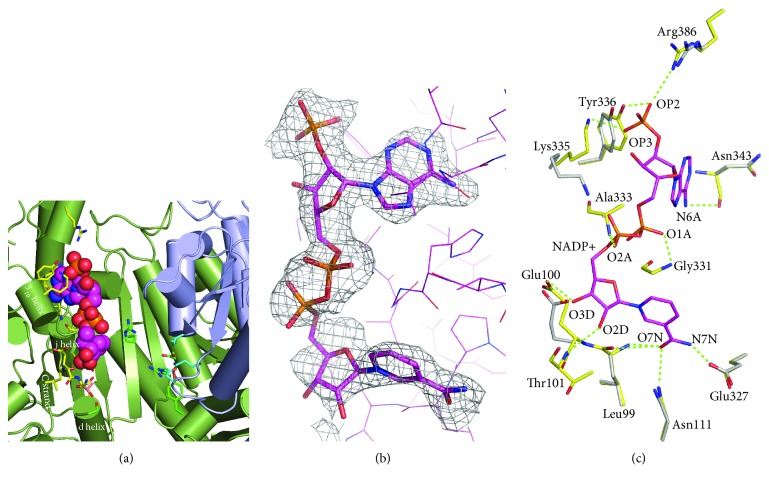
View around the binding site of NADP^+^. (a) View around the active site cleft. NADP^+^ is drawn in a sphere model. NADP/putative isocitrate bound residues are shown in a stick model. Thr101 is colored in pink. Polypeptide chains are drawn in a cylinder model. (b) Omit maps of NADP^+^. Maps are contoured at 1.6 *σ* superimposed with the final model. (c) View of the NADP^+^-binding site. Residues in the apo and holo forms are colored in white and yellow, respectively, and superimposed. Oxygen, nitrogen, and sulfur atoms are shown in red, blue, and orange, respectively. Dotted lines indicate hydrogen bonds.

NADP^+^ is bound with the residues derived from the large domain at the upper part of the open active site cleft, apart from the postulated isocitrate-binding site, suggesting that the substrate access to its binding site may not be limited (Figure 4(a)). The electron density maps clearly revealed that the NADP^+^ molecule adopts a U-shaped conformation, in which the adenine ring and the nicotinamide ring are in the anticonformation with respect to each adjacent ribose (Figure 4(b)). The adenine ring is hydrogen bonded with the main chain oxygen of Asn343 in the Dj loop (N6A–O). In the deep cleft, the nicotinamide ring interacts with the side chains of Asn111 in the Cd loop (O7N–ND2) and Glu327 in the carboxyl terminal of the D strand (N7N–OE1) and with the main chain of Leu99 in the C strand (O7N–N/O). The adjacent ribose is hydrogen bonded to the side chain of Glu100 (O3D-OE1) and the main chain nitrogen of Thr101 (O2D–N) both in the Cd loop. The Cd loop running along NADP^+^ is sandwiched by these adenine and nicotinamide rings of NADP^+^, and the diphosphate group is hydrogen bonded to the main chain nitrogens of Gly331 (O1A–N) and Ala333 (O2A–N) in the central part of this loop. These binding residues with NADP^+^ described above in the holo form of ST2166 are almost identical in location and orientation to those of the apo form except for Thr101, which is disordered in the apo form (Figure 4(c)).

**Figure 5:**
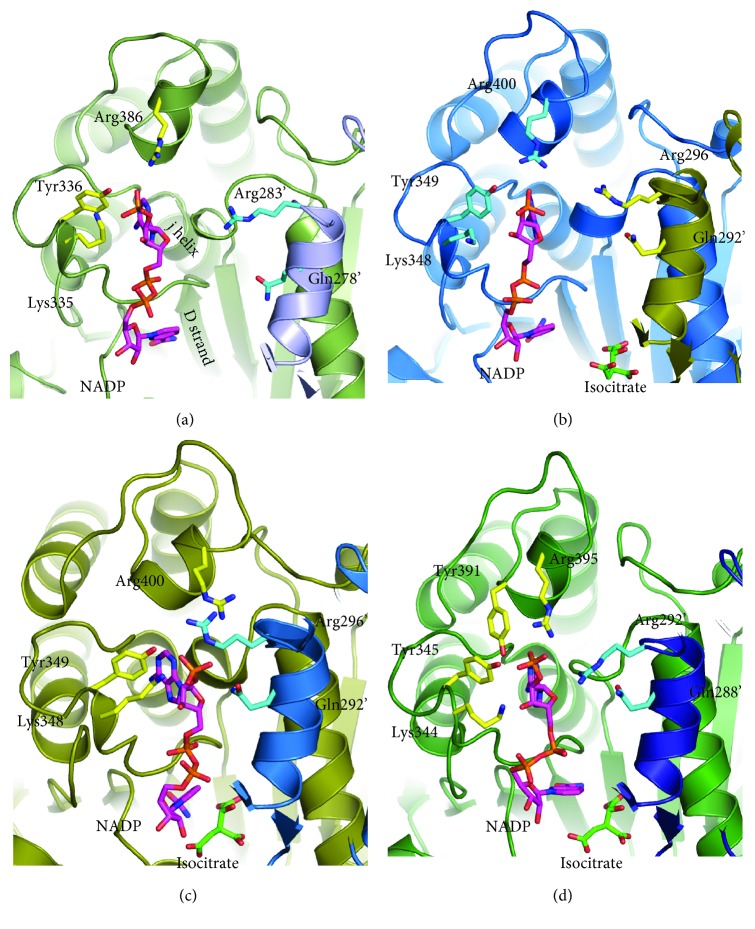
Structural comparisons of the binding site of the 2′-phosphate group of NADP^+^ between ST2166, ApIDH, and EcIDH. (a–d) View of the binding site of ST2166 (a), ApIDH in the open form (b), ApIDH in the closed form (c), and EcIDH in the closed form (d), respectively. Polypeptide chains are drawn in a ribbon model. NADP^+^, isocitrate, and residues are shown in a stick model. Oxygen, nitrogen, and sulfur atoms are shown in red, blue, and orange, respectively. Red dotted lines indicate hydrogen bonds.

The 2′-phosphate group of NADP^+^ is bound to the side chains of Lys335 (OP3–NZ), Tyr336 (OP2–OH), and Arg386 (OP2–NH1). Because NAD^+^ possesses a hydroxyl group at the 2′-moiety, these direct interactions of the large domain with the 2′-phosphate group of NADP^+^ from a single subunit are responsible for the discrimination of NADP^+^ from NAD as a cofactor in the open form of ST2166. It is noteworthy that the former three residues are from a short 3_10_ helix formed in the Dj loop and the latter is from the l helix (Figures 4(a) and 5(a)). Therefore, it is likely that they function as a rigid binding site for the 2′-phosphate group of NADP^+^.

### 3.5. Insights into the Cofactor Recognition

Lys335, Tyr336, and Arg386 interacted with the 2′-phosphate group of NADP^+^ in ST2166 (Figure 5(a)). In the open subunit of the ternary complex of ApIDH, the 2′-phosphate group of NADP^+^ is indeed interacted with the corresponding Tyr349 and Arg400 and Lys348 is situated close (6.2 Å) to it (Figure 5(b)). As these residues are completely conserved among prokaryotic NADP-IDHs, this binding mode can play an important role in specifying the mode of cofactor recognition.

On the other hand, the manner of binding of the 2′-phosphate group of NADP^+^ is different in closed conformations. In the closed subunit of the ternary complex of ApIDH, the 2′-phosphate group of NADP^+^ interacts with the side chains of Tyr349, Gln292′, and Arg296′ (corresponding to Tyr336, Gln279′, and Arg283′ in ST2166, respectively) at the active site (Figure 5(c)). EcIDH forms a closed conformation with NADP^+^ and isocitrate, in which Tyr345, Tyr391, and Arg395 (corresponding to Tyr336, Gln382, and Arg386 in ST2166, respectively) participate to bind with the 2′-phosphate group of NADP^+^ (Figure 5(d)). These observations show that binding residues in the closed form are somewhat different between the organisms. These interactions were summarized in Table 2.

It is interesting to note that the backbone helical structure in the Dj loop corresponding to the 3_10_ helix in ST2166 is often found in NADP-IDHs of subfamily II. IDH from eukaryote *Saccharomyces cerevisiae* belonging to subfamily II interacts with the 2′-phosphate group of NADP^+^ by Arg316 and His317 (corresponding to Lys335 and Tyr336 in ST2166). The residues are from *α*11, which is proposed to act as a lid in the closed form by covering the active site cleft [31]. Comparisons between the structures of IDHs from thermophilic eubacteria *Clostridium thermocellum* and psychrophilic *Desulfotalea psychrophila* show a locking mechanism of the large domain to the small domain by moving the helix module including *α*11 [32, 33]. Therefore, the helix structures in the Dj loop likely participate in regulating the enzymatic activity upon closure of the active site in subfamily II IDHs. For prokaryotic NADP-IDHs, the 3_10_ helix in the Dj loop found in ST2166 is also formed in ApIDH, but not in EcIDH (Figure 5). It is known that AceK, which regulates the catalytic activity by phosphorylation on Ser113 in EcIDH, only works in gram-negative bacteria, but not in either *S. tokodaii* or *A. pernix* due to the absence of structural elements required for AceK recognition [34–36]. Therefore, these helical structures in ST2166 and ApIDH may be related to the regulation mechanism in closed conformations such as those observed in subfamily II IDHs.

The apo crystal of ST2166 was easily cracked upon soaking with citrate but not NADP^+^, suggesting that citrate mimicking the substrate isocitrate can bind to the opening active site of the enzyme and induce a large conformational change to break the crystal lattice. Therefore, binding of NADP^+^ is not necessary for a large domain movement in ST2166. It is also conceivable that the binding of isocitrate or NADP^+^ is independent to each other in other NADP-IDHs, including ApIDH and EcIDH. However, some interactions between NADP^+^ and isocitrate have been observed via the phosphorylation loop of the enzymes in the structures complexed with them. In the closed subunit of ApIDH, the nicotinamide ribose interacts with the main chain nitrogen of Thr112 (O3D–N) as well as the *γ*-carboxyl group of isocitrate (O2D–O3). The *γ*-carboxylate group of isocitrate is also close to hydrogen bond to the side chain of Thr112 (O3–OG1). In the open subunit of ApIDH, the side chain of Thr112 interacts with the ribose, as seen in corresponding Thr104 in the closed form of EcIDH (O2D–OG1). These structures indeed contain NADP^+^ and isocitrate in both the open and closed active sites; however, the nicotinamide moiety of NADP^+^ is disordered. Instead, fully ordered electron densities for bound NADP^+^ are observed in a pseudo-Michaelis complex of EcIDH with NADP^+^, isocitrate, and Ca^2+^ in the closed form, where Ca^2+^ acts as a competitive inhibitor for Mg^2+^/Mn^2+^ [7, 37]. Given that the main chain of Thr101 makes a hydrogen bond to the nicotinamide ribose of NADP^+^ with the side chain directing toward the putative isocitrate-binding cavity in the holo form of ST2166, it is suggested that NADP^+^ should already be recognized within the active site and proceed to domain closure for effective catalytic activity.

The 7-fold mutant Cys201Met/Cys332Tyr/Lys344Asp/Tyr345Ile/Val351Ala/Tyr391Lys/Arg395Ser of EcIDH shows the conversion of the cofactor specificity from NADP^+^ to NAD^+^, which was governed by the interactions of the 2′-hydroxyl group of NAD^+^ with the positions 344, 345, 391, and 395 [38]. Because Tyr391 of EcIDH is occupied by Gln in both ST2166 and ApIDHs and not conserved in prokaryotic NADP-IDHs, these residues might not be involved in the interaction with NADP^+^. The other three residues at positions 344, 345, and 391 correspond to Lys335, Tyr336, and Arg386 in ST2166 and bind to the 2′-phosphate group of NADP^+^, and these residues are completely conserved in prokaryote NADP-IDHs including ApIDH and EcIDH. Therefore, it is conceivable that the cofactor selectivity in prokaryote NADP-IDHs is determined by the three residues Lys335, Tyr336, and Arg386 in ST2166 binding to the 2′-phosphate group of NADP^+^ in the open form, not in the closed form.

## 4. Conclusion

In this study, the putative IDH from *S. tokodaii*, ST2166, was crystallized in the apo and NADP^+^-bound forms. Because ST2166 shows high (~50%) identities of the amino acid sequence with those of other prokaryotic NADP-dependent IDHs, EcIDH, and ApIDH, common features associated with the cofactor selectivity were investigated. The structures showed that NADP^+^ molecule can bind tightly at the opening active site in ST2166 and Lys335 and Tyr336 and Arg386 interacted with the 2′-phosphate moiety of NADP^+^. Because these residues are completely conserved between prokaryotic NADP-IDHs, we propose that Lys335, Tyr336, and Arg386 in ST2166 are essential for cofactor recognition and bind NADP^+^ before the domain closure in prokaryotic IDHs.

## Figures and Tables

**Table 1:** Data collection and final refinement statistics.

	Apo form	NADP^+^-bound form
Data collection		
Wavelength (Å)	1.0	1.0
Resolution (Å) (outer shell)	2.0 (2.11–2.00)	2.40 (2.53–2.40)
Completeness (%) (outer shell)	99.5 (99.8)	98.8 (100)
No. of observed reflections	234,961	132,519
No. of unique reflections	65,823	38,351
Multiplicity	3.6 (3.5)	3.5 (3.6)
*R*_sym_^1^ (%) (outer shell)	6.2 (41.3)	8.4 (37.3)
Space group	P21	P21
Unit cell parameters (Å, °)	*a* = 74.88	*a* = 74.78
	*b* = 87.72	*b* = 88.11
	*c* = 75.72	*c* = 75.67
	*α* = *γ* = 90.00	*α* = *γ* = 90.00
	*β* = 91.37	*β* = 91.36
Refinement		
Resolution (Å)	15.00–2.00	15.00–2.40
Number of protein atoms	6418	6446
Number of water molecules	463	405
Number of NADP^+^ atoms	0	96
*R*_work_^2^ (%)	22.1	21.7
*R*_free_ (%)	24.7	27.9
RMS deviation bond length (Å)	0.0059	0.0071
RMS deviation bond angle (°)	1.264	1.396
Average B-factor (Å)	34.09	34.88

^1^ *R*_sym_ = ∑_hkl_∑_*i*_|*I*_*i*_ − 〈*I*〉|/∑_hkl_∑_*I*_*I*_*i*_, where *I*_*i*_ is the intensity of an individual reflection and 〈*I*〉 is the mean intensity obtained from multiple observations of symmetry-related reflections. ^2^*R*_work_ = ∑_hkl_(*F*_obs_ − |*F*_calc_|)/∑_hkl_|*F*_obs_| (9.2% randomly omitted reflections were used for the calculation of *R*_free_).

**Table 2:** Possible hydrogen bonds of the 2′-phosphate moiety.

	(Å)
ST2166 (open)	
OP3–Lys335 (NZ)	2.8
OP2–Tyr336 (OH)	2.8
OP2–Arg386 (NH1)	3.1
ApIDH (open)	
OP2–Tyr349 (OH)	3.6
OP2–Arg400 (NH1)	3.1
ApIDH (closed)	
OP1–Lys348 (NZ)	4.0
OP3–Tyr349 (OH)	2.5
OP2–Arg296′ (NH1)	3.4
OP2–Gln292′ (NE2)	3.9
EcIDH (closed)	
OP1–Tyr345 (OH)	2.6
OP3–Tyr391 (OH)	2.7
OP2–Arg395 (NH1)	3.0
OP2–Arg395 (NH2)	2.5
OP2–Arg395 (NH2)	2.6

Distances of less than 4.0 Å between hydrogen bond partners of the 2′-phosphate moiety of NADP^+^ and IDH.

## Data Availability

Coordinates and structural parameters had been published with Protein Data Bank under accession code 2E0C for the apo form and 2E5M for the NADP^+^-bound form.
